# Effects of Pre-Stretching on the Mechanical Behavior of Cold-Rolled 5%Mn Medium Manganese Steel

**DOI:** 10.3390/ma16216926

**Published:** 2023-10-28

**Authors:** Lifeng Fan, Yulong Yang, Jiao Huang, Erbin Yue, Wenhao Hu, Jun Gao

**Affiliations:** 1 School of Materials Science and Engineering, Inner Mongolia University of Technology, Hohhot 010051, China; fanlifeng829@163.com (L.F.); yangyulong_995@163.com (Y.Y.); 2 Zhejiang Metallurgical Research Institute Co., Ltd., Hangzhou 310005, China; yuewheat@126.com (E.Y.); huwenhao@hzsteel.com (W.H.); 3 Inner Mongolia Baotou Steel Union Co., Ltd., Baotou 014010, China; laogao032149@163.com

**Keywords:** medium manganese steel, austenite, strength–ductility combination, pre-stretching

## Abstract

Studies about the pre-stretching effect on the mechanical behavior of cold-rolled 5%Mn medium manganese steel have adopted optical microscopy, scanning electron microscopy, transmission electron microscopy and X-ray diffraction techniques. Results showed that pre-stretching would change the ferrite morphology from massive and lath-like to strip-like. With the pre-stretching increasing from 0% to 14%, the dislocation density and yield strength both grew gradually, which corresponded to growth from 6.49 × 10^14^ m^−2^ to 7.98 × 10^14^ m^−2^ and growth from 765 MPa to 1109 MPa, respectively. Meanwhile, the austenite volume fraction, elongation and product of strength and elongation were all reduced with the pre-stretch increase. The stabilized retained austenite with pre-stretch delayed the occurrence of the TRIP effect and improved the work hardening rate. As a result, the Lüders band disappeared at 2% pre-stretch and the PLC band vanished from the stress–strain curve at 14% pre-stretch.

## 1. Introduction

With the implementation of “carbon peak, carbon neutralization”, light weight automobiles and indicators of energy saving, emissions reduction and safety have become increasingly prominent for automobile steel production. Studies showed that there was, simultaneously, an 8% reduction in vehicle fuel consumption, 6% reduction in carbon dioxide emissions and 4% reduction in other gas emissions with a 10% reduction in vehicle weight [1]. Advanced high-strength steel (AHSS) for automobiles was called for to meet this demand. At present, the third-generation automobile steel with an M3 microstructure, which is multiphase (ultra-fine grain ferrite, martensite and austenite), metastable (formation of retained austenite and solid solution strengthening of the alloy), multi-size (grain size, lath width and stacking fault microstructure control) and controlled with heat treatment of reverse phase transformation annealing, is a current research hotspot [2,3].

There was a phenomenon of plastic instability on the third-generation automobile steel such as the yield platform and serrated fluctuation to the yield point. The plastic instability not only affected the surface quality of the material, but also adversely affected the plasticity of the material, and became a key problem restricting the mass production and wide application of medium manganese steel. Li et al. [4] showed that the yield platform gradually decreased on the engineering stress–strain curve until it disappeared with the increase in the pre-strain variables. An opposite result by Zhang et al. [5] was that a yield platform was on the stress–strain curve with the increase in the pre-strain variables. Li et al. [6] found that the product of strength and plasticity reached the maximum under a 16% pre-deformation of a 2050Al-Li alloy, but a further increase in the pre-deformation to 20% made the strength decrease. Tian et al. [7] found that 5~16% pre-stretching deformation extended the fatigue life of a 7N01 aluminum alloy by over 23%, but the pre-stretching deformation increased to 20%, and a further increase in the pre-deformation to 20% made the elongation decrease from 23.2% to 5.2% and the fatigue life shorten.

Some retained austenite in the third-generation automobile steel could significantly improve the strength and toughness of the material. The deformation of retained austenite induces martensite transition; that is, the phase-change-induced plasticity (TRIP) effect could reduce the local stress concentration and delay the formation of microcracks, which is the key factor to improve the plasticity of Q and P steel [8]. Qin et al. [9] showed that the mechanism of high fatigue performance for high-carbon Q-P-T steel mainly stemmed from two aspects: one was the dislocation absorption of the retained austenite (DARA) effect existing in the fatigue test, which significantly enhanced the deformation ability of the martensite matrix, and the other was the deformation-induced martensitic transformation effect, which could effectively arrest the crack to combat fatigue. Therefore, it is necessary to study the phase transition amount of retained austenite during the deformation process.

To study the effects of the pre-stretching amount and austenite volume on the mechanical properties and microstructure evolution of 5% Mn medium manganese steel, the experiment in this study was conducted with a two-step uniaxial tension test, which consisted of the first loading under various pre-stretching amounts and second loading until it was broken. The Lüders band and PLC (Portevin–Le Chatelier) effects which were eliminated using pre-stretching and the elimination of the plastic instability phenomenon during the deformation are discussed.

## 2. Material and Experimental Procedures

In total, 43.5 kg of a pure iron rod with 99.5% purity and C content less than 0.04% was melted using a 50 kg vacuum smelting furnace with a vacuum degree less than 20 Pa and temperature higher than 1600 °C. The crucible of the furnace was cleaned to remove residual slags to prevent impurities from affecting the purity of experimental steels before melting. Pure metals of alloying elements were added into the liquid iron using the hopper in the furnace and then evenly melted. A quartz glass sampler was used for a sample analysis of the liquid experimental steel. The chemical composition of steel samples was determined with an ARL-4460 spectrometer (Hebei iron and steel technology research institute, Shijiazhuang, China). Composition-qualified liquid steel that was superheated from 50 °C to 60 °C was poured directly into a casting mold of heat-resistant cast iron in the vacuum furnace. Then, it was cooled in the induction furnace under the vacuum. The composition of the experimental steel is shown in Table 1.

The size of the ingot was 150 mm (thickness) × 150 mm (width) × 250 mm (length). The ingot was hot-rolled from 150 mm to 5 mm with a Φ750 × 550 mm two-roll experimental mill after homogenization at 1150 °C, and the final rolling temperature was 800 °C. The hot-rolled plates were water-cooled to 300 °C with laminar flow and then were air-cooled to room temperature. The plates were cold-rolled to 1.5 mm using a four-high direct-pull reversible cold rolling mill.

The critical temperatures on heating, i.e., AC1 (austenite transformation beginning temperature during calefaction) and AC3 (austenite transformation finish temperature during calefaction) of the test steel, measured using a DSC differential thermal analyzer (Setaram in Lyon, France, Labsys Evo TG-DSC), were 591 °C and 736 °C, respectively. The samples were quenched after heating at 930 °C for 20 min and were annealed with reverse phase transformation at 675 °C for 30 min in a bright annealing furnace. The gauge distance is 50 mm of standard tensile samples according to the national standard GB/T228-2002 [10] samples. The two-step uniaxial tension test was carried out by a WDW-300/100 G microcomputer-controlled electronic universal testing machine (Changchun new testing machine Co., Ltd., Changchun, China). The first loading speed was 2 mm min^−1^, and the pre-stretching strain was 0%, 2%, 5%, 7%, 12%, 14% and 17%, respectively. The second loading speed was the same as the first one and pre-stretching samples were broken at room temperature.

Microstructures of samples with various pre-stretching were observed for studying microstructure evolution of the experimental steel by a GX51 F optical microscope (Carl Zeiss, Oberkochen, Germany), a FEI QUANTA650 scanning electron microscope (FEI, Production in Czech Republic, USA brands) and a Tecnai G2F20 Transmission Electron Microscope (FEI, Hillsboro, OR, USA) [11]. C and Mn element distribution was detected by a JXA-8530F Plus electron probe (JEOL Ltd., Akishima, Japan). The sample for OM and SEM observation was made into 10 mm (ND) × 15 mm (RD) metallographic sample by wire cutting. The metallographic sample was set with an XQ-1 metallographic sample machine(Shanghai Jinxiang Machinery & Equipment Co., Ltd., Shanghai, China), and the setting powder was phenolic plastic powder. The embedded sample was polished step by step with sandpaper ranging from 120 mesh to 2000 mesh. Then, the grounded sample was polished by a PG-1 metallographic sample polishing machine (Shanghai Jinxiang Machinery & Equipment Co., Ltd., Shanghai, China). The 3.5 μm polishing paste was used to polish the surface to a mirror. The sample was finally etched with a 4% nitrate alcohol solution, rinsed with alcohol and dried before OM and SEM observation. The working parameters of the SEM were a voltage of 15 kV, a current of 6μA and a point resolution of 1.3 nm. Samples for TEM observation were mechanically ground to 30~50 µm and punched into a size of Ф3 mm. Then, the sample was polished and etched in an electrolyte of 4% HClO_4_ alcohol solution by a MTP-AStruers twin-jet electro-polisher(Shanghai Jiaoda Electromechanical Technology Development Co., LTD., Shanghai, China). The double spray voltage ranged from 30 V to 50 V, the current was controlled at 25 mA. The double spray temperature was controlled at −20 °C by liquid nitrogen.

The austenite content in steel was analyzed by an X Pert PRO MPD X-ray diffractometer (PANalytical B.V., Almelo, The Netherlands). Three austenite γ phase diffraction lines of (200 )γ, (220)γ and (311)γ and two martensite α phase diffraction lines of (200)α and (211)α were scanned, and the corresponding diffraction angle 2θ and cumulative strength were measured to calculate the volume fraction of retained austenite using Equation (1) [12]:

(1) $$V_{\gamma }=\frac{1.4I_{\gamma }}{I_{\alpha }+1.4I_{\gamma }}\;$$

*V_γ_* is the volume fraction of austenite; *I_γ_* is the integral intensity of the diffraction peak of the austenite crystal plane; *I_α_* is the integral intensity of the diffraction peak of the ferrite crystal plane.

## 3. Results and Discussion

**Figure 1:**
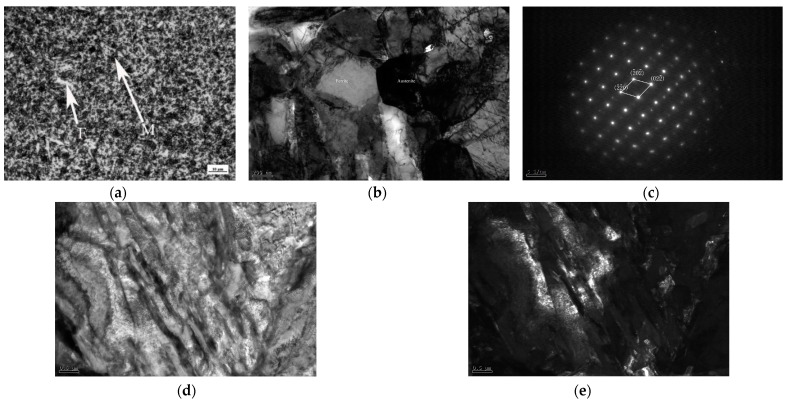
Microstructure of the experimental steel after reverse phase transformation annealing at 675 °C for 30 min. (**a**) OM; (**b**) TEM; (**c**) SAED patterns of the austenite; (**d**) bright field image; (**e**) dark field image corresponding to Figure 1d.

**Figure 2:**
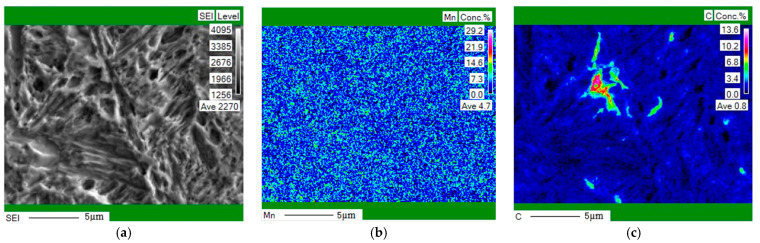
EPMA mapping of C and Mn element distribution in experimental steel after reverse phase transformation annealing at 675 °C for 30 min. (**a**) Scanning area; (**b**) distribution of Mn; (**c**) distribution of C.

The microstructure of the steel after reverse phase transformation annealing is as shown in Figure 1 [13]. Our previous experimental study showed that the microstructure consisted of ultrafine grained ferrite, martensite, and 22.34% retained austenite after austenitizing at 930 °C for 20 min then quenching, and reverse phase transformation annealing at 675 °C for 30 min [14,15,16]. After annealing at 675 °C, the carbides were nearly completely dissolved, providing sufficient energy for austenite nucleation [14,15,16]. During the annealing process in the two-phase region, C and Mn elements could fully diffuse from ferrite to austenite and stabilize the retained austenite. However, the average concentration of C and Mn elements in the austenite would decrease resulting from the growth of grain size and the amount of austenite with the increase in annealing temperature. Some unstable austenite transformed into ferrite and martensite during cooling leading to decrease in the austenite volume fraction. Figure 2 showed that the distribution of Mn was more uniform than that of C, which locally reached 10.2%, far beyond the 0.13% of the uniform experimental material. It indicated that C had a more noticeable effect on stabilizing austenite.

**Figure 3:**
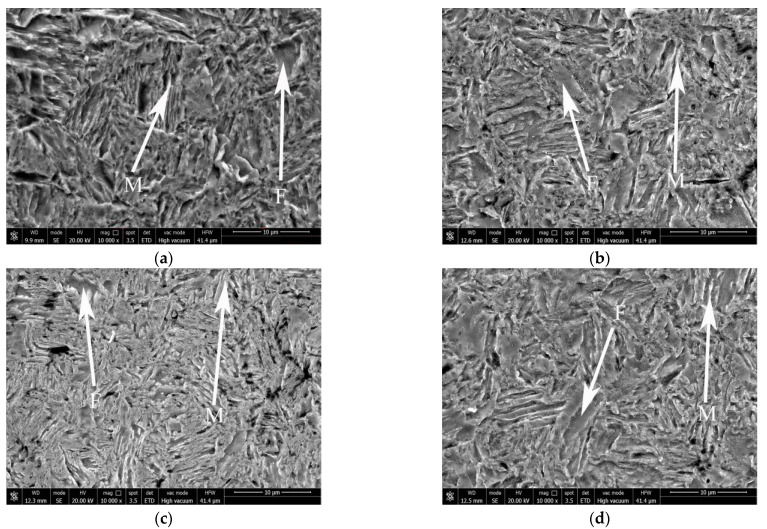
Microstructure of experimental steel under various pre-stretching amounts after reverse phase transformation annealing at 675 °C for 30 min. (**a**) 0%; (**b**) 2%; (**c**) 7%; (**d**) 14%.

Figure 3 was the microstructure of the experimental steel samples corresponding to the pre-stretching amount of 0%, 2%, 7% and 14%. It could be observed that the microstructure consisted of concave block or plate ferrite, protruding lath martensite and austenite. When the pre-stretching amount was less than 7%, the ferrite in the experimental steel evolved from block to strip with the increase in the pre-stretching amount. When the pre-stretching amount reached 14%, part of the ferrite changed from a concave shape to a protruding shape. The deformation of ferrite was caused by squeezing during the transformation of austenite to martensite under the TRIP effect during the stretching process [17].

**Figure 4:**
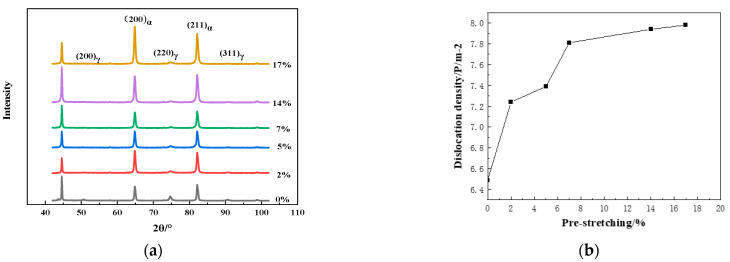
XRD diffraction spectra and dislocation density in ferrite under various pre-stretching amounts. (**a**) XRD diffraction spectrum, (**b**) dislocation density in ferrite.

Another significant change in the microstructure was the growth in dislocation density with the increase in the pre-stretching amount. Figure 4 shows the XRD diffraction spectrum of the experimental steel (a) and dislocation density in ferrite (b) under various pre-stretching amounts. The dislocation density in ferrite was calculated by Equation (2) [18,19], where ρ is the dislocation density, β is the full width at half maximum of the (211)α diffraction peak of ferrite, b is the Burgers vector mode of the material, with a value of 2.48 × 10^−10^ m [20,21]:

(2) $$\rho =\frac{\beta^{2}}{4.35{\times b}^{2}}$$

It can be seen that the dislocation density increased with the rise in the pre-stretching amount. The dislocation density in ferrite increased from 6.49 × 10^14^ m^−2^ to 7.98 × 10^14^ m^−2^ with the pre-stretching amount increasing from 0% to 17%. When the austenite changed to martensite under the TRIP effect during tensile deformation, the newly formed martensite produced an extrusion against the ferrite surrounding it, further increasing the dislocation density. 

**Figure 5:**
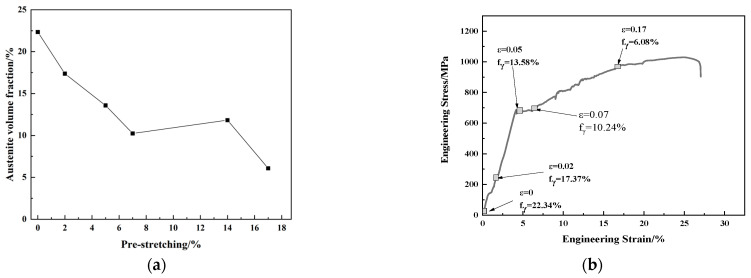
Stress–strain curve and austenite volume fraction of cold rolled medium manganese steel after reverse phase transformation annealing at 675 °C for 30 min. (**a**) Volume fraction of austenite; (**b**) stress–strain curve.

Figure 5a shows the austenite volume fraction of the cold-rolled medium manganese steel after reverse phase transformation annealing at 675 °C for 30 min with various pre-stretching amounts. With the increase in the pre-stretching amount, the volume fraction of austenite decreased except for the deformation with a 14% pre-stretching.

The volume fraction of austenite in the experimental steel was 22.34% without pre-stretching. When the pre-stretching amount increased to 2%, the volume fraction of austenite decreased to 17.37%. It indicated that the mechanical stability of some austenite was poor and could transform to martensite under a tiny stress at the initial stage of deformation [22]. With the increase in strain, the volume fraction of austenite decreased obviously. Combined with Figure 5b, it showed that a discontinuous TRIP effect occurred at the deformation ranging from 7% to 17%. The transformation of austenite phase into martensite phase during deformation enhanced the work hardening, relieved the stress concentration, delayed the crack generation, and improved the plasticity of the material. When the deformation strain was 17%, the volume fraction of austenite was only 6.08%, less than 8% which was the minimum austenite content for an obvious TRIP effect. Therefore, the work hardening curve happened at stage III. At this time, the microstructure of the experimental steel was composed mainly of ferrite and new martensite.

With the increase in the pre-stretching amount from 0% to 7%, the austenite content decreased from 22.34% to 10.04%. However, the austenite content decreased from 10.04% to 6.08% with the pre-stretching amount increase from 7% to 17%. Thus, the austenite TRIP effect mainly occurred before a 7% pre-stretching. From the relationship between dislocation density and pre-stretching amount presented in Figure 4b, it can be seen that the increment of dislocation density in ferrite after pre-stretching deformation from 7% to 14% was much lower than that in ferrite after pre-stretching from 5% to 7%. As mentioned above, the dislocation increment was mainly caused by the TRIP effect. Therefore, the dislocation increment mainly occurred after a pre-stretching less than 7% and the austenite content changed slightly after a 7% pre-stretching.

**Figure 6:**
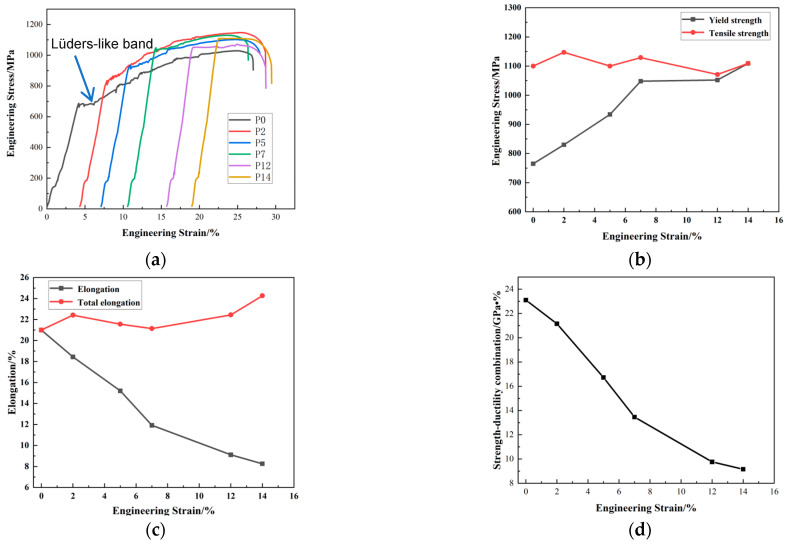
Mechanical properties of cold-rolled medium manganese steel annealed at 675 °C for 30 min under various pre-stretching amounts. (**a**) stress–strain diagram; (**b**) yield strength and tensile strength; (**c**) elongation; (**d**) strength-ductility combination.

The engineering stress–strain curves of the cold-rolled medium manganese steel sample after reverse phase transformation annealing with various pre-stretching amounts was showed in Figure 6a. The yield platform and jagged fluctuation on the stress–strain curve of the test steel without pre-tension illustrated a plastic instability phenomenon. This jagged fluctuation on the tensile stress–strain curve was called the PLC effect, which was caused by dynamic strain aging (DSA) [23]. The PLC effect is a typical plastic instability phenomenon of materials under a certain loading strain rate and experimental temperature. The PLC effect was detrimental to the surface quality of the material during processing or in service and to the plasticity and formability of the material, even to the fatigue cycle and restricted the service life of the material. The yield point disappeared under a 2% pre-stretching amount. The frequency and amplitude of jagged fluctuation were reduced with the increasing in the pre-stretching amount until it completely disappeared under a 14%pre-stretching.

The mechanical properties of the experimental steel under different pre-stretching amounts are presented in Figure 6b–d. With the increase in the pre-stretching amount, the yield strength increased, the elongation and product of strength and elongation decreased, and the tensile strength fluctuated in the range of 1071 MPa to 1147 MPa.

The yield strength increased from 765 MPa to 1109 MPa with the pre-stretching amount increasing from 0% to 14%. The yield strength increased sharply under a pre-stretching less than 7%, and rose slowly under a pre-stretching over 7%. Considering that the dislocation density in ferrite grew up with the increase in the pre-stretching amount, the volume fraction of the retained austenite decreased after pre-stretching, and the volume fraction of transformed martensite rose. It could be inferred that the increase in yield strength was mainly due to dislocations increasing in the ferrite [24].

The total elongation was a sum of the two-step tension test, which was the total of the pre-stretching amount under the first loading and elongation under the second loading on each sample. As illustrated in Figure 6c, the pre-stretching amount increased from 0% to 14%, the elongation (the second loading) decreased from 21% to 8.26%. The total elongation increased with the increase in the pre-stretching amount, and reached 24.26% with a 14% pre-stretching amount. The product of strength and elongation gradually decreased from 23.1 GP·% without pre-stretching to 9.16 GP·% with a 14% pre-stretching, as shown in Figure 6d. With the increase in the pre-stretching amount, the retained austenitic volume fraction in the steel was gradually reduced (Figure 5a), which decreased the TRIP effect and the second elongation rate.

The similar trend of elongation and austenite volume fraction with the pre-stretching amount further proved that elongation was affected by the austenite volume fraction. Meanwhile, the increase in dislocation density and phase transition martensite volume fraction could also compromise the plasticity, reduce the elongation rate and strength-ductility [25].

**Figure 7:**
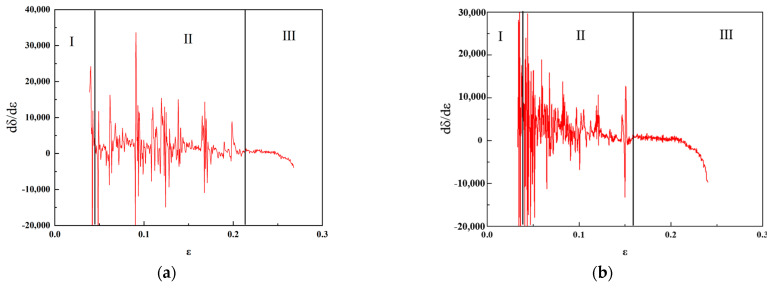

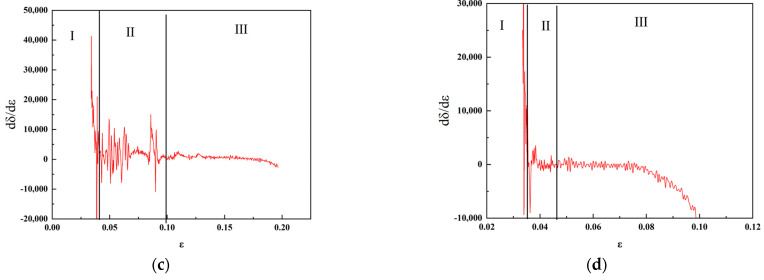
Work-hardening curves of experimental steel under various pre-stretching amounts (**a**) 0%; (**b**) 2%; (**c**) 5%; (**d**) 14%.

Work hardening curves of the experimental steel under different pre-stretching amounts are shown in Figure 7, exhibiting a typical three stages curve. The first stage was characterized by a decreasing fluctuation state. The second stage showed a fluctuating state due to the discontinuous TRIP effect. The strain length, fluctuation amplitude and fluctuation period became smaller with the increase in the pre-stretching amounts. When the pre-stretching amount was 2%, the TRIP effect of austenite and the enhancement of strain hardening ability shortened the fluctuation period and increased the work hardening rate. When the pre-stretching amount was 5%, the austenite conversion rate before and after stretching decreased. The stability of retained austenite increased, which weakened the TRIP effect, making the second stage smooth further. When the pre-stretching amount was 14%, the volume fraction of austenite continuously decreased, the TRIP effect was almost negligible. It could be concluded that a small pre-stretching amount enhanced the TRIP effect but reduced the volume fraction of austenite in the experimental steel. While a relative large pre-stretching amount made the austenite too stable to induce the TRIP effect. In the third stage, the work hardening rates decreased slowly until fracture.

**Figure 8:**
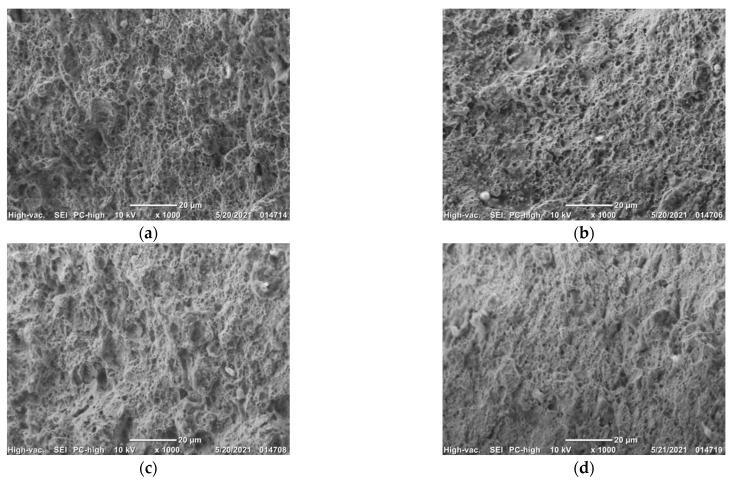
Fracture morphology after application of various pre-stretching amounts (**a**) 0%; (**b**) 2%; (**c**) 7%; (**d**) 14%.

The fracture morphology of the cold-rolled medium manganese steel under various pre-stretching amounts is presented in Figure 8. The number and density of dimples decreased with the increase in the pre-stretching amount indicating a plasticity decrease in test steels. The fracture of the sample without pre-stretching (Figure 8a) was ductile with more dimples and a higher dimple density. When the pre-stretching amount was 14%, nearly no dimples were observed at the fracture morphology, showing a brittle fracture.

The Lüders-like bands on yield point appeared on the stress–strain curve of the experimental steel after reverse phase transformation annealing, which vitally affected the surface quality of the steel sheets. Thus, the surface of the automobile steel was good enough before stamping, but would become corrugated or rough after stamping.

Li [4] and Wang [26] also found that the yield point gradually decreased with the increase in the pre-stretching amount, and disappeared when the pre-stretching amount was equal to the length of the yield platform. The generation of Lüders bands was caused by dislocation slip and the Cottrell pinning effect produced by solute atoms (such as C and N atoms) at dislocations [27,28]. In the medium manganese steel with the TRIP effect, the generation of the Lüders band was a result of the combination of the TRIP effect and dislocation slip [25], which was affected by the austenite volume fraction, stability and work hardening rate [29,30,31]. In this study, the yield point on the stress–strain curve after 2% pre-stretching disappeared, as shown in Figure 6a. The volume fraction of austenite decreased to 17.37% which stabilized the retained austenite. The work hardening rate was positively correlated with the austenite amount and would be enhanced by the dislocation accumulation and martensitic transformation during the tensile deformation. The dislocation density in ferrite increased with the rise in pre-stretching amount (Figure 4b) and reached a considerable amount under a 2% pre-stretching. In addition, the deformation-induced martensite acted as a second phase to hinder dislocation movement [32]. The work hardening effect would be enhanced at the effect of the dislocation increment and the dislocation movement hindrance during the pre-stretching contributed to the multifaced functioning enhancement of the austenite stability, dislocation density and work hardening rate in ferrite, and the Lüders bands were eliminated. Therefore, a certain amount of pre-stretching could be performed on the parts to eliminate surface defects caused by the Lüders bands in actual production.

Dislocation movements would be hindered by various obstacles such as precipitates and grain boundaries and be restarted by stress over a pinning force. The process of repeated pinning and de-pinning was characterized by jagged features on the stress–strain curve. The dislocation of the steel would be split into a head dislocation, an extended dislocation and a tail dislocation. When the extended dislocation moved, it would run through the C-Mn atom pair and bring out the C atom. The C atom would drag the tail dislocation when returned to an octahedral gap. The repeating process of dragging and detaching of an extended dislocation by the C atom would form a serrated stress which was manifested as the PLC effect [33]. The microstructure of the experimental steel with reverse phase transformation annealing comprised ultrafine-grained ferrite, austenite and martensite which was partly transformed by a strain-induced effect during the deformation process.

Due to the redistribution behavior of C and Mn during heat treatment, the C content in the ferrite of medium manganese steels was too low to produce stacking faults with extended dislocations [34]. Meanwhile, the ferrite did not produce the PLC effect at room temperature. The martensite was a hard phase with limited deformation. So, the PLC effect in medium manganese steels was neither related to the ferrite nor to the martensite. The austenite was prone to dislocations and it was easy to produce the PLC effect through the C atoms vibration and the interaction of stacking faults [35,36].

It can be seen in Figure 6a that the stress–strain curve of the experimental steel shows a ‘linear’ continuous yield characteristic with a 12% and 14% pre-stretching amount, especially for a 14% pre-stretching. The difference was that there were not any obvious upper and lower yield points on the platform compared with the yield platform of the stress–strain curve without pre-stretching. Cheng et al. [37] found that if the grain size was 0.1 μm, the dislocation storage in grains was limited; if the grain size was larger than 1 μm, dislocations were generated at grain boundaries and within grains, and improved the work hardening ability of grains. The DSA generation needs sufficient dislocation density and solute atoms. After annealing, numbers of the C-Mn atom pairs in the medium manganese steel were sufficient. However, a large amount of unstable austenite preferentially transformed into martensite during the pre-stretching process, resulting in a gradual decrease in the remaining austenite grain size. The smaller was the austenite grain size, the lower was the dislocation storage capacity. Thus, the dislocation multiplication rate was reduced by the deformation, resulting in insufficient dislocations stored in metastable austenite grains until necking to produce PLC bands. When the pre-stretching amount stood at 14%, the PLC bands in the stress–strain curve of the experimental steel disappeared, showing a continuous yield phenomenon.

## 4. Conclusions

With the increase in the pre-stretching amount, shapes of ferrite changed from block and lath to slender strip form, and the dislocation density in ferrite increased from 6.49 × 10^14^ m^−2^ to 7.98 × 10^14^ m^−2^. The volume fraction of the austenite in the experimental steel decreased from 22.34% to 6.08% with the rise in the pre-stretching amount, and the stability of austenite increased.

With the increase in the pre-stretching amount under 14%, the yield strength of the experimental steel increased from 765 MPa to 1109 MPa, the tensile strength fluctuated in a range of 1071 to 1147 MPa, the elongation and the product of strength and elongation gradually decreased, but the total of the pre-stretching amount and elongation increased slightly. The fracture evolved from ductile with uniform distribution of dimples to brittle fracture.

The Lüders bands appeared in the tensile process of the experimental steel without pre-stretching. When the pre-stretching amount was 2%, the Lüders bands disappeared due to the multifaced functioning enhancement of austenite stability, dislocation density and the work hardening rate in ferrite.

The PLC effect in the medium manganese steel was produced in the retained austenite, and the PLC band disappeared on the stress–strain curve after 14% pre-stretching, showing a continuous yield.

## Figures and Tables

**Table 1:** Chemical composition of experimental steel (mass fraction/%).

C	Mn	Cu	Al	Ni	Nb	Ti
0.13	5.4	0.24	0.043	0.24	0.032	0.017

## Data Availability

No new data were created or analyzed in this study. Data sharing is not applicable to this article.
